# Analysis of the Components in Moxa Smoke by GC-MS and Preliminary Discussion on Its Toxicity and Side Effects

**DOI:** 10.1155/2020/2648759

**Published:** 2020-10-31

**Authors:** Xiaoyu Xu, Si Shan, Wenlei Wang, Hongning Liu

**Affiliations:** ^1^Jiangxi Province Key Laboratory of TCM Etiopathogenisis, Jiangxi University of Traditional Chinese Medicine, Nanchang, Jiangxi 330004, China; ^2^Research Center for Differentiation and Development of TCM Basic Theory, Jiangxi University of Traditional Chinese Medicine, Nanchang, Jiangxi 330004, China

## Abstract

Moxibustion plays an important role in the prevention and treatment of diseases and the promotion of human health. In this study, the components in moxa smoke from Jiangxi Poai Biotechnology Co., Ltd., namely, Qing moxa sticks, were absorbed by five solvents (cyclohexane, ethyl acetate, *n*-butanol, anhydrous ethanol, and water) and identified by gas chromatography-mass spectrometry. The identification results of the smoke from the Qing moxa sticks that was absorbed in liquid are as follows: a total of 294 compounds were identified, including 139 in cyclohexane, 145 in ethyl acetate, 60 in *n*-butanol, 89 in anhydrous ethanol, and 77 in water, and of those, 112 toxic compounds were identified. Furthermore, Ingenuity Pathway Analysis software and the PubChem database were successfully applied to analyze the toxic compounds. There were 812 target proteins related to the toxic components, 25 molecular networks, and 54 biological pathways. The results showed that the toxic compounds of moxa smoke may have some side effects on the heart, liver, and kidney of humans. This study revealed that the components of moxa smoke are complex and diverse. Due to the findings of toxic compounds in moxa smoke, we recommend that moxibustion rooms should be equipped with ventilation equipment or enough artificial ventilation to ensure the health of patients and practitioners.

## 1. Introduction

Moxibustion is an important part of clinical treatment in traditional Chinese medicine. In moxibustion, wormwood or other drugs are used to place acupoints or pain points on the body surface for warming meridians and stimulating acupuncture points [1]. As people pay more attention to health, the use of moxibustion to treat diseases in China and other Asian countries is growing [2]. Heat and moxa smoke are produced during moxibustion. The heat of moxibustion has the function of assisting Yang Qi, lifting subsidence, and solidifying. Recent studies have shown that moxa smoke also has antibacterial, antitumor, antiviral, anti-inflammatory, and air purification functions [3–7]. Ancient books on Chinese Medicine contain records of the use of moxa smoke in the treatment of irritable bowel syndrome [8], inflammatory bowel disease [9], and neurological symptoms [10]. Additionally, the antioxidants in moxa smoke play an antiaging role through the penetration of heat [11].

However, some patients feel uncomfortable during moxibustion and can even have noticeable adverse reactions, such as watery eyes and coughing, which has caused people to question the safety of moxa smoke [12, 13]. Some studies have shown that there were harmful components such as monoaromatic hydrocarbons and formaldehyde in moxa smoke [14–16]. The inhalation of these substances induced eustachian tube irritation, throat itching, eye pain, tonsil swelling, and other toxic effects [12]. Therefore, it is very important to determine the toxic compounds in moxa smoke.

The aim of the present study was to analyze the components in Qing moxa smoke based on enrichment with five solvents. A set of smoke absorption devices were designed with cyclohexane, ethyl acetate, *n*-butanol, anhydrous ethanol, and water as absorbents with the help of an extraction pump to concentrate the moxa smoke in the solvents. The benefits of this device for enrichment of moxa smoke include: (1) moxa sticks can burn completely in the air to avoid incomplete combustion; (2) the devices can detect as many compounds as possible by increasing the concentration of moxa smoke; and (3) the use of different polar solvents can provide reference for the absorption and treatment of moxa smoke. Then, the toxic compounds were queried by the Comparative Toxicogenomics Database (CTD) [17, 18]. In addition, we aimed to estimate the toxic compounds in moxa smoke that would have an impact on the human body by applying Ingenuity Pathway Analysis (IPA) software and the PubChem database to provide an experimental basis for the safety evaluation of moxa smoke [19, 20].

## 2. Materials and Methods

### 2.1. Materials

We followed the steps outlined in our patent, “A method of using Terahertz Wave to detect the quality of moxa column,” patent number: ZL 2020 1 0000161.6, which are as follows: (1) sample placement; (2) determination of the background value; (3) measurement of the terahertz wave energy at different bands of the combustion column; (4) data processing; and (5) column quality judgment. If the terahertz wave intensity of each band is stronger than others and the waveform slightly changes, the quality is better. The results revealed which Qing moxa stick had the best quality, and that one was selected for smoke enrichment analysis [21]. Qing moxa sticks (18 × 27 ± 1 mm, Jiangxi Poai Biotechnology Co., Ltd., Poyang, China), which are widely used by the Chinese population, were used in this study. Moxa sticks were encased in *Artemisia argyi* (Chinese mugwort) floss, which was made of dried *A. argyi* leaves. The Qing moxa sticks were produced with a 10 : 1 ratio, which means that 10 kg of dried *A. argyi* leaves were processed into 1 kg of moxa floss. Analytical grade cyclohexane, ethyl acetate, *n*-butanol, and ethanol were all purchased from Guangdong Xilong Science Co., Ltd., and used as received.

### 2.2. Sample Preparation

**Figure 1:**
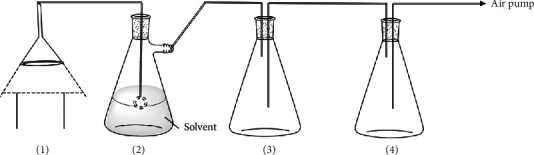
A smoke absorption device. (1) Smoke hood. (2) Absorption flask. (3) Blank flask. (4) Buffer flask.

A set of smoke absorption devices was designed as shown in Figure 1. With 1000 mL cyclohexane, ethyl acetate, *n*-butanol, anhydrous ethanol, or water as the solvent, 50 moxa sticks were burned in the air until combustion was complete. During the combustion process, the air pump control combustion speed was adjusted such that the blank flask did not fill with white smoke, so that the solvent fully absorbed the moxa smoke. The glass ball in the absorption flask had holes in it to reduce the production of bubbles and prevent the solvent from escaping. The absorption solution was emptied from the absorption flask, filtered with a 0.22 *μ*m microporous membrane, and 2 mL of each solution was added into the sample bottle for gas chromatography-mass spectroscopy (GC-MS) analysis.

### 2.3. GC-MS Analysis

An Agilent Technologies 7890 GC system (Agilent Technologies Inc., Palo Alto, CA, USA) coupled with an Agilent Technologies 5975 mass spectrometer (Agilent Technologies Inc.) was used for moxa smoke analysis. A HP-5MS capillary column (30 m × 0.25 mm × 0.25 *μ*m) was used to separate compounds. High-purity helium was applied as the carrier gas. The following conditions were used: column flow rate: 1.0 mL/min; split injection, split ratio: 100 : 1; injection volume: 1 *μ*L; and injection port temperature: 250°C. The temperature procedure was as follows: 0–3 min, 40–40°C; 3–39 min, 40–220°C; 39–43 min, 220–220°C; 43–49 min, 220–280°C; and 49–50 min, 280–280°C.

The MS working conditions were as follows: the electron ionization energy was 70 eV, the full-scan acquisition was used in the range of 50–650 m/z, the ion source temperature was 230°C, the transmission ion temperature was 280°C, and the four-stage pole temperature was 150°C. The identification of each peak in the total ion flow chromatogram was automatically retrieved from the National Institute of Standards and Technology (NIST) 11.L as the standard mass spectrometry database and verified with standard mass spectrometry. Some components were confirmed with the retention value of a standard sample. The identified components were semiquantified by comparing the peak area of each component with the total peak area, and the relative percentage of components was calculated by the peak area normalization method.

### 2.4. Network Toxicological Analysis

The compounds identified by the NIST 11.L were then queried for related toxicity through the CTD database (https://ctdbase.org/about/). Then, the molecular information corresponding to the toxic compounds of moxa smoke was obtained from the PubChem database (http://pubchem.ncbi.nlm.nih.gov/) [22]. In addition, the Swiss Target Prediction database (http://www.swisstargetprediction.ch/) was used to predict toxic compounds relevant targets, and exporting Uniprot ID. Next, the molecular networks of toxic compound target proteins and its biological pathways were constructed by IPA software (Qiagen, Redwood City, CA, USA).

## 3. Results

### 3.1. Total Ion Chromatogram

**Figure 2:**
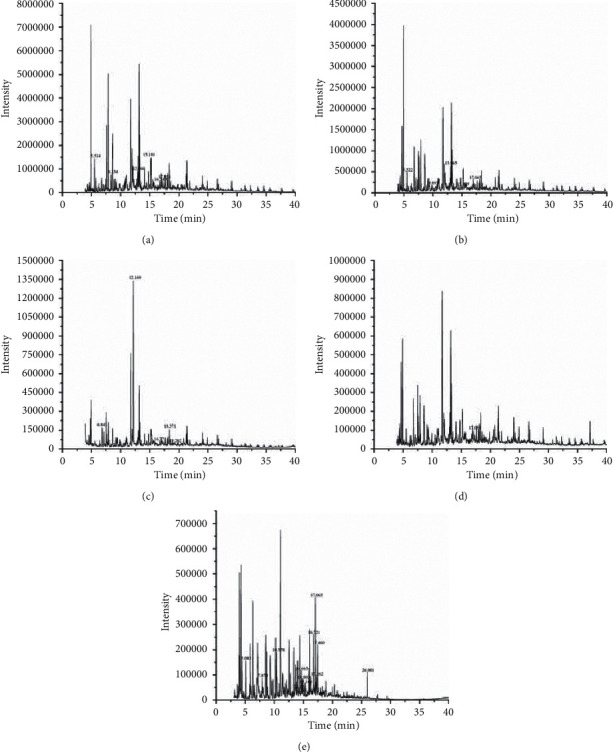
Total ion chromatograms of five solvents by GC-MS. (a) Cyclohexane. (b) Ethyl acetate. (c) *n*-Butanol. (d) Anhydrous ethanol. (e) Water. Only compounds unique to each solvent with a relative content greater than 0.5% are tagged in the figure.

The total ion chromatograms (TIC) of moxa smoke from solvents by GC-MS are shown in Figure 2 [23, 24]. As shown in Figure 2, the compounds in moxa smoke were detected within 40 min.

### 3.2. GC-MS Analysis Results

**Figure 3:**
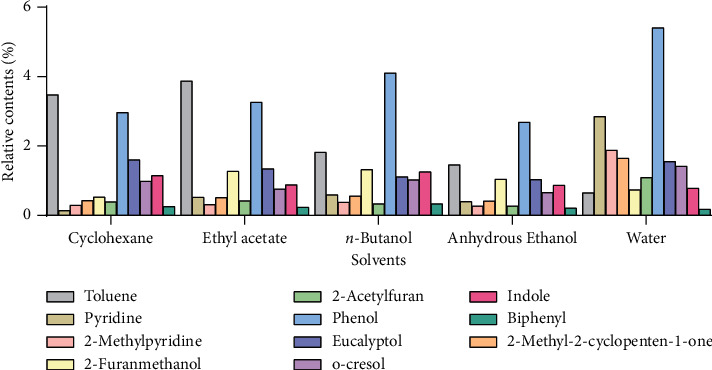
Relative contents (%) of common compounds in the five solvents.

A total of 294 compounds, including 139 in cyclohexane, 145 in ethyl acetate, 60 in *n*-butanol, 89 in anhydrous ethanol, and 77 in water were found and identified in Qing moxa smoke. As shown in Tables 1–5, only 52 unique compounds were detected in cyclohexane smoke absorption liquid, 57 in ethyl acetate, 10 in *n*-butanol, 17 in anhydrous ethanol, and 47 in water, and other components were identified in more than one solvent. Toluene, pyridine, 2-methylpyridine, 2-methyl-2-cyclopenten-1-one, 2-furanmethanol, 2-acetylfuran, phenol, eucalyptol, o-cresol, indole, and biphenyl were detected in all five solvents and are shown in Figure 3, but the same components had different concentrations in different solvents. This shows that the components of moxa smoke were absorbed differently by different polar solvents.

As shown in Figure 3, the common components from moxa smoke in the five solvents included toluene (0.650%–3.872%), pyridine (0.137%–2.847%), 2-methylpyridine (0.267%–1.878%), 2-methyl-2-cyclopenten-1-one (0.412%–1.649%), 2-furanmethanol (0.526%–1.320%), 2-acetylfuran (0.266%–1.092%), phenol (2.686%–5.405%), eucalyptol (1.037%–1.605%), o-cresol (0.661%–1.419%), indole (0.780%–1.257%), and biphenyl (0.179%–0.338%). Among the above common components, the relative contents of phenol were more than 2% in all solvents. Phenol is a corrosive compound that is a strong irritant, which can lead to acute poisoning, skin ulcers, and tissue burns and can even be life-threatening [25, 26]. However, the amount of harmful substances produced by moxibustion will dictate the negative impact on the human body, and the duration of exposure to a moxa fume environment will determine if damage is caused to the body. There is no unified answer to these questions, which requires a large amount of case analysis and clinical trials.

### 3.3. Toxic Compounds of Moxa Smoke

The toxicity of compounds was determined based on the CTD database (https://ctdbase.org/about/), which provided abundant toxicological information for researchers. Among the 294 compounds detected in the moxa smoke absorption liquid, 112 compounds were confirmed to be toxic. Further study is needed to explore the toxicity of the 112 compounds.  Table 6 provides details of the 112 toxic compounds.

### 3.4. Targets of Toxic Compounds

Through the PubChem database (http://pubchem.ncbi.nlm.nih.gov/), molecular information for the 112 toxic compounds in moxa smoke was identified, and the corresponding number of “Canonical SMILES” was obtained. Then, using the Swiss Target Prediction database (http://www.swisstargetprediction.ch/) to predict the 112 relevant targets of the toxic compounds, the UniProt ID was exported. In addition, the UniProt ID was analyzed with IPA software to obtain the targets of toxic compounds. There were 812 targets for the toxic compounds in moxa smoke, compared to 810 identified with the IPA database.

### 3.5. Molecular Networks of Toxic Compounds

**Figure 4:**
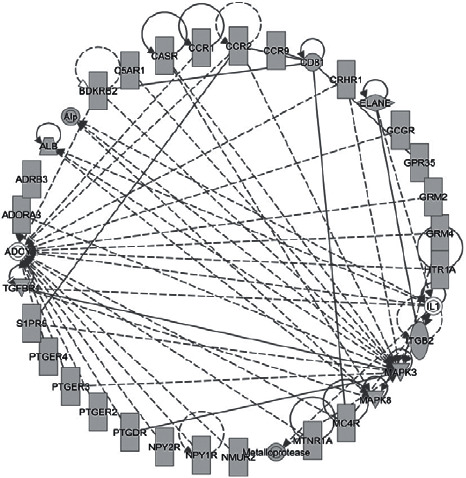
Molecular network with a maximum score of 43. Each node in the figure represents 1 molecule, the solid lines represent a direct interaction between two molecules, and the dotted lines represent an indirect interaction between two molecules.

The UniProt IDs of the 810 target proteins of the 112 toxic compounds were imported into the IPA bioanalysis software. Under the “tox analysis” module, IPA was used to construct the molecular networks of target proteins. A total of 25 molecular networks were constructed for 112 toxic compounds, with a maximum score of 43, as shown in Figure 4. The results showed that these target proteins were related to cell signal transduction, nucleic acid metabolism, inflammatory response, organ damage, and cell apoptosis. Therefore, this can be used to frame a correlation study on moxa smoke.

### 3.6. Biological Pathways of Toxic Compounds

**Figure 5:**
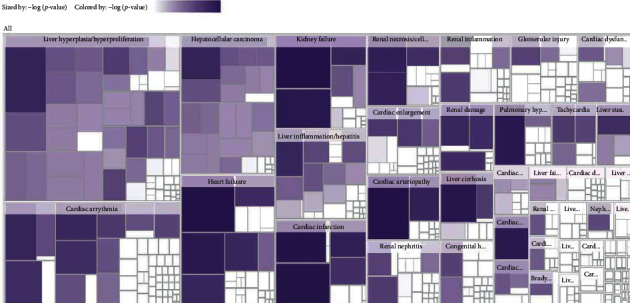
A heat map of the biological pathway of toxic compounds. The pathway scores are displayed using a purple color gradient, where darker purple corresponds to higher scores (increased statistical significance).

Using the “tox analysis” module in the IPA software, a total of 54 biological pathways were found for the 112 toxic compounds. The main biological pathways of the toxic compounds from moxa smoke included cardiotoxicity, hepatotoxicity, and nephrotoxicity. Consequently, the toxic compounds of moxa smoke may have some side effects on the human heart, liver, and kidneys. A heat map of the biological pathway of toxic compounds is shown in Figure 5. According to it, the pathway with the highest −log (*p* value) was cardiac arteriopathy, which was classified as cardiotoxicity, with a value of 79.429. Drug target molecules acting on this pathway include ABCB1, ABCC8, ACE, ADORA1, ADORA2A, ADORA2B, ADORA3, ADRA2A, ADRA2B, ADRA2C, ADRB1, ADRB2, ADRB3, ALDH5A1, ALOX5AP, AR, ASIC3, CA1, CA12, CA13, CA14, CA2, CA3, CA4, CA5A, CA5B, CA6, CA7, CA9, CACNA2D1, CETP, CNR1, CYP2C19, CYP2C9, DPP4, ESR1, ESR2, F10, F2, F2R, FADS1, FKBR1A, FLT1, FLT4, GAA, GABRA1∗, GABRA2∗, GABRA3∗, GABRA5∗, GABRB2∗, GABRB3∗, GABRG2∗, GLP1R, GLRA1, GRIA4, HCAR2, HMGCR, HRH2, HTT, ICAM1, INSR, ITGAL, ITGB2, KCNA5, KCNJ11, KDM1A, KDR, MTNR1A, MTNR1B, MTOR, NOS3, NPC1L1, NR3C1, NR3C2, OPRD1, OPRK1, OPRM1, PDE10A, PDE11A, PDE3A, PDE3B, PDE4A, PDE4B, PDE4C, PDE4D, PDE5A, PDE7A, PDE7B, PGR, PLA2G2A, PLA2G7, PLG, PPARA, PPARG, PRCP, PRKCH, PTGER1, PTGER2, PTGER3, PTGER4, PTGIR, PTGS1, PTGS2, RHOA, S1PR1, SCARB1, SCN10A, SCNSA, SCN9A, SELE, SERPINE1, SLC6A4, SOAT1, TBXA2R, TERT, TLR4, TNF, TNNT2∗, TSPO, TUBB1, TUBB3, VDR, VEGFA, and XDH. This also guides the development of follow-up toxicology experiments and research on the effects of moxa smoke on the organs of Sprague Dawley rats.

## 4. Discussion

GC-MS was applied to study the compounds in moxa smoke absorbed in five different polar solvents from Qing moxa sticks. This study found that a total of 294 compounds were identified, including 139 in cyclohexane, 145 in ethyl acetate, 60 in *n*-butanol, 89 in anhydrous ethanol, 77 in water, and 11 in all five polar solvents. Among the 294 compounds detected in the moxa smoke absorption liquid, 112 compounds were confirmed to be toxic. With the “tox analysis” module, IPA was used to construct molecular networks of target proteins. The results showed that these target proteins were related to cell signal transduction, nucleic acid metabolism, inflammatory response, organ damage, and cell apoptosis. At the same time, the main biological pathways of the toxic compounds from moxa smoke included cardiotoxicity, hepatotoxicity, and nephrotoxicity. The safety of smoke has become greater concern. The question of whether moxa smoke is harmless or not has become key to restricting the use of moxibustion.

At present, most studies on moxa smoke have shown that it has many pharmacological effects. A study [27] showed that the superoxide anion scavenging activity of moxa smoke was superoxide dismutase 24.4 U/mg, which was slightly higher than that of partially purified moxa extract and alkali-lignin, but lower than that of sodium ascorbate, gallic acid, and catechin, which further confirmed the antioxidant and pro-oxidative effects of moxa smoke. The methanol extract of moxa smoke has the functions of antioxidation and eliminates free radicals [28]. Another study demonstrated that moxa smoke can improve sperm concentration and promote sperm movement in rats [29]. Although it was suggested that the toxic compounds in moxa smoke were harmful to the human heart, liver, and kidneys, low and middle concentrations had no effects. Moxa smoke at higher concentrations might destroy heart, liver, and kidney function. In fact, it has been reported that moxa smoke can cause related symptoms, such as eustachian tube and throat itching, eye pain, tonsil enlargement, and other symptoms [30–33]. Tar contains two-tenths of a million of a kind of thick cyclic aromatic hydrocarbon called benzo(a)pyrene, which is a strong carcinogen [34]. In a few cases, patients undergoing moxibustion treatment or after treatment had erythema, blisters, and other hypersensitive symptoms, and these conditions disappeared after leaving the moxa smoke environment [35–37]. Research results show that moxibustion may have a greater impact on some people with chronic pharyngitis, leading to coughing due to moxa smoke allergy, but these symptoms gradually improved after ventilation [38]. Some scholars have placed rats in a dynamic exposure cabinet and observed the content of Ox-LDL in their serum. The results showed that the content of Ox-LDL decreased gradually with the increase of moxa smoke concentration, suggesting that moxa smoke can reduce the degree of platelet aggregation. Therefore, it may improve microcirculation and promote metabolism of the body. Low concentrations of moxa smoke have no noticeable damage to vascular endothelium, while medium concentrations can cause a certain degree of vascular endothelium damage [39, 40].

The moxa sticks were encased in *A. argyi* floss, which is made of dried *A. argyi* leaves. There have been many experimental studies on the toxicity of *A. argyi*, which were not limited to conventional acute toxicity, subacute toxicity, or chronic toxicity. Domestic scholars have conducted in-depth studies on the hepatorenal toxicity, embryonic toxicity, and genetic toxicity caused by *A. argyi*. The research objects were not limited to the whole animal, but also extended to the cellular level, and the intrinsic mechanism of some toxicity of *A. argyi* was also discussed. The relationship between quantity, time and toxicity, and a safe time span for use were also discussed. However, some of the results showed that *A. argyi* had hepatotoxicity, especially the essential oil of *A. argyi* [41, 42]. The dosage of *A. argyi* or moxa sticks used in toxic experiments was more than 10 to 200 times the clinical dosage. According to the results of this paper, we carried out toxicological experiment of moxa smoke in rats. We followed the steps outlined in our patent, “A device for enriching moxa smoke and its analytical method,” patent number: CN202010327163.6 [43]. Rats exposed to 756650 mg/m^3^ concentration of moxa smoke (concentration of moxa smoke in 50 moxa sticks) were compared with the control group, and the structure of myocardial cell, hepatic cell, and the renal tubules showed changes (Supplementary Figure S1) such as cardiac hypertrophy, degeneration and necrosis, and dilatation of renal tubules, respectively.

In a word, we should not discuss the toxicity in terms of toxicity in isolation but should comprehensively consider the clinical use characteristics of traditional Chinese medicine. However, in clinical application, we should pay attention to its “toxicity” to human body and try to avoid overuse. Therefore, moxibustion rooms should have installed ventilation equipment or the room should have adequate artificial ventilation so that the health of patients and practitioners can be guaranteed. The safety of compounds in moxa smoke needs to be further studied. The results of this study provide a basis for a safety evaluation of moxa smoke in the future.

## Figures and Tables

**Table 1:** Relative content (%) of unique compounds in cyclohexane.

No.	Rt (min)	Compound	Retention index	Relative content (%)
1	4.038	Bicyclo[4.1.0]hept-2-ene	706	0.033
2	4.602	4-Methyl-1,4-hexadiene	737	0.175
3	4.978	1-Methylcyclohexene	757	0.078
4	5.093	1,3,5-Heptatriene	763	0.115
5	5.290	1,7-Octadiene	774	0.048
6	5.400	2-Methyl-1-heptene	780	0.105
7	5.514	1-Octene	786	0.693
8	5.947	(E)-2-Octene	806	0.086
9	6.458	1,3-Dimethyl cyclohexene	822	0.222
10	6.647	(E,E,E)-2,4,6-Octatriene	828	0.027
11	7.111	5,6-Dimethyl-1,3-cyclohexadiene	843	0.040
12	7.749	3-Methylenecycloheptene	863	0.146
13	8.060	(1Z,2Z)-1,2-Di(ethylidene)cyclobutane	873	0.094
14	8.334	Cyclohexanol	882	0.703
15	9.025	2-Ethylpyridine	904	0.095
16	10.064	2-Methyl-1-octen-3-yne	935	0.105
17	10.166	1-Methylcycloheptene	938	0.141
18	11.164	Mesitylene	968	0.307
19	11.629	Alpha-methyl styrene	981	0.176
20	12.372	Gamma-terpinene	1004	0.227
21	12.960	1,2,4-Trimethylbenzene	1022	0.866
22	13.395	Trans-beta-methyl styrene	1035	0.371
23	14.973	1-Phenyl-2-butene	1083	0.228
24	15.181	1-Methyl-4-(prop-1-en-2-yl)benzene	1090	1.054
25	15.901	Cosmene	1112	0.163
26	16.210	2,4-Dimethylstyrene	1122	0.156
27	16.305	1-Phenyl-1-butene	1125	0.336
28	16.448	1-Allyl-2-methylbenzene	1130	0.223
29	16.749	Phenyl acetonitrile	1140	0.610
30	17.051	2,3-Dimethylphenol	1149	0.687
31	17.155	1,2,3,4-Tetramethylbenzene	1153	0.203
32	17.510	1,1a,6,6a-Tetrahydrocycloprop[a]indene	1164	0.258
33	18.241	1,2-Dimethylindan	1188	0.200
34	19.970	4-Methylindole	1247	0.200
35	20.792	7H-Benzocycloheptene	1275	0.200
36	21.045	2-(2-Hydroxyphenyl)buta-1,3-diene	1284	0.168
37	22.396	1H-Indene,2,3-dihydro-1,1,3-trimethyl	1332	0.226
38	23.401	5-Methylindole	1369	0.099
39	23.527	1,8-Cyclotetradecadiyne	1374	0.108
40	24.816	1,4-Dimethylnaphthalene	1422	0.227
41	25.073	1,4,5-Trimethylnaphthalene	1432	0.142
42	26.434	2,4,6-Trimethylbenzonitrile	1485	0.137
43	27.199	1-Phenylpyridin-2-one	1516	0.186
44	28.473	2,4-Dimethoxyacetophenone	1568	0.234
45	28.910	Spathulenol	1586	0.163
46	29.355	2-Methylbiphenyl	1605	0.114
47	30.735	(+)-*γ*-Gurjunene	1665	0.217
48	33.434	9-Methylene-9H-fluorene	1786	0.103
49	34.460	3,7,11,15-Tetramethyl-2-hexadecene	1835	0.062
50	34.710	2,6,10,14-Tetramethyl-2-hexadecene	1847	0.135
51	35.671	1-Nonadecene	1893	0.163
52	37.669	E-15-Heptadecenal	1993	0.123

**Table 2:** Relative content (%) of unique compounds in ethyl acetate.

No.	Rt (min)	Compound	Retention index	Relative content (%)
1	4.106	3-Methyl-butanenitrile	710	0.159
2	4.535	Dimethyl aminoacetonitrile	733	0.191
3	4.603	2,4-Dimethyl-1,3-pentadiene	737	0.152
4	5.236	3-Methylenecyclohexene	771	0.095
5	5.300	Cyclooctene	775	0.030
6	5.522	2-Octene	787	0.557
7	5.837	2,3-Dimethyl-1,4-hexadiene	802	0.120
8	5.957	(Z)-2-Octene	806	0.044
9	6.462	Pyrazine, methyl	822	0.208
10	6.869	2,5-Dimethylpyrrole	835	0.088
11	8.073	1,4-Dimethylenecyclohexane	874	0.051
12	8.411	2,3-Dimethylpyridine	885	0.166
13	9.820	3-Ethyl-1H-pyrrole	928	0.295
14	9.893	3,4-Dimethylpyridine	930	0.509
15	10.078	2-Ethyl-5,5-dimethyl-1,3-cyclopentadien	935	0.082
16	10.480	2-Methylborazine	947	0.250
17	11.291	2,5-Cyclooctadien-1-one	971	0.115
18	11.406	Benzene	975	0.060
19	11.503	Aniline	978	0.183
20	12.166	3-Methylstyrene	997	0.337
21	12.385	Alpha-phellandrene	1004	0.151
22	12.591	2-Ethyl-4-methyl-1H-pyrrole	1010	0.295
23	13.065	o-Cymene	1025	1.118
24	13.402	Allylbenzene	1035	0.309
25	15.039	3-Ethyl-o-xylene	1085	0.593
26	15.620	7-Methylbenzofuran	1103	0.383
27	15.921	Azulene	1113	0.406
28	16.454	4-Allyltoluene	1130	0.171
29	16.762	3-Ethynylaniline	1140	0.367
30	17.065	3-Methyl-1H-indene	1150	0.557
31	17.163	1,2,3,4-Tetramethylfulven	1153	0.138
32	17.523	1,4-Dihydronaphthalene	1165	0.214
33	18.254	1-Methyl-3-(1-methyl-2-propenyl)benzene	1188	0.188
34	18.501	Dihydrocarveol	1196	0.140
35	18.696	Catechol	1203	0.223
36	19.034	2,6-Dimethylundecane	1215	0.195
37	19.250	(E)-Cinnamaldehyde	1222	0.391
38	19.355	Cyclododecene	1226	0.166
39	19.758	Isoquinoline	1239	0.210
40	19.983	3-Methylindolizine	1247	0.172
41	21.057	1,11-Dodecadiene	1284	0.162
42	22.733	2-Methylhydroquinone	1345	0.174
43	22.816	Naphthalene, 1,2,3,4-tetrahydro-1, 1-dimethyl	1348	0.125
44	23.825	2-Methyl-5-(1-methylethenyl)-cyclohexanone	1385	0.131
45	23.955	3-Methylindole	1389	0.257
46	24.827	1,3-Dimethylnaphthalene	1423	0.205
47	24.930	1,6-Dimethylnaphthalene	1427	0.460
48	25.761	2-Phenyl-1,3-cyclohexadien	1459	0.104
49	28.833	Phenylephrine	1583	0.020
50	31.836	Thiazolo[5,4-f]quinolin	1713	0.158
51	32.239	1,1,2-Trimethylcycloundecane	1732	0.250
52	33.442	Phenanthrene	1787	0.116
53	34.719	3,7,11,15-Tetramethyl-2-hexadecene	1847	0.146
54	35.480	(S)-6,6-Dimethyl-2-azaspiro[4.4]non-1-ene	1884	0.158
55	37.681	3-Icosene	1994	0.179
56	39.610	10-Heneicosene (c,t)	2093	0.140
57	39.729	Heneicosane	2100	0.110

**Table 3:** Relative content (%) of unique compounds in *n*-butanol.

No.	Rt (min)	Compound	Retention index	Relative content (%)
1	6.843	3-Furaldehyde	834	1.167
2	9.850	3-Methylheptan-4-one	928	0.377
3	9.998	2,4-Dimethylpyridine	933	0.457
4	10.819	Limonene	957	0.442
5	12.169	Butyl butyrate	997	6.894
6	16.330	(E)-1-Phenyl-1-butene	1126	0.273
7	16.771	Benzyl(methylidyne)azanium	1140	0.559
8	18.371	cis-2-dodecene	1192	1.064
9	19.262	Tricyclo[3.3.1.0(2,8)]nona-3,6-dien-9-one	1222	0.600
10	23.958	1-Methylindolizine	1390	0.361

**Table 4:** Relative content (%) of unique compounds in anhydrous ethanol.

No.	Rt (min)	Compound	Retention index	Relative content (%)
1	5.142	Thiophene	766	0.064
2	6.488	2-Methylpyrazine	823	0.137
3	9.811	2,3-Dimethyl-1H-pyrrole	927	0.155
4	11.170	6-Methyl-6-ethylfulvene	968	0.191
5	12.724	Acrylamide	1014	0.098
6	14.877	(−)-Camphor	1080	0.477
7	14.974	2-Methyl-1-phenylpropene	1083	0.137
8	15.691	4-Pyridinol	1105	0.157
9	17.059	3-Phenyl-1,2-butadiene	1150	0.649
10	17.518	Benzo[2,3]bicyclo[3.1.0]hexane	1165	0.188
11	20.713	Citral	1272	0.283
12	21.053	4-Methyl-2H-benzopyrane	1284	0.160
13	22.331	1,7-Dimethylnaphthalene	1330	0.224
14	25.053	2,3,6-Trimethylnaphthalene	1432	0.123
15	29.065	(Z)-8-Hexadecene	1593	0.393
16	35.803	Nonadecane	1900	0.118
17	37.154	Dibutyl phthalate	1968	0.425

**Table 5:** Relative content (%) of unique compounds in water.

No.	Rt (min)	Compound	Retention index	Relative content (%)
1	4.688	Methallyl cyanide	741	0.065
2	5.083	Cyclopentanone	763	0.730
3	5.558	Tetrachloroethylene	789	0.076
4	6.010	4-Aminopyridine	808	0.470
5	6.479	2-Methylcyclopentanone	823	0.375
6	6.549	4-Methylpentanenitrile	825	0.314
7	6.679	(R)-(+)-3-Methylcyclopentanone	829	0.150
8	7.870	2,6-Dimethylpyridine	867	0.596
9	8.091	Cyclohexanone	874	0.378
10	8.420	5,5-Dimethyl-1,3-hexadiene	885	0.079
11	8.816	2-Ethylpyrazin	898	0.426
12	8.975	2,3-Dimethylpyrazine	903	0.126
13	10.000	2,5-Dimethylpyridine	933	0.497
14	10.378	5-Methylfurfural	944	1.092
15	11.178	Phenetole	968	0.185
16	11.346	1-Isopropylcyclopentene	973	0.312
17	12.121	2-Ethyl-6-methylpyridine	996	0.438
18	12.400	2-Ethyl-5-methylpyridine	1004	0.282
19	12.687	5-Ethyl-2-methylpyridine	1013	0.207
20	12.743	2-Acetyl-5-methylfuran	1015	0.095
21	13.570	1-Acetyl-2-methyl-1-cyclopentene	1040	0.378
22	14.229	2-Methyl-6-methylene-2,7-octadien-4-ol	1060	0.540
23	14.663	Sabinene hydrate	1074	0.750
24	14.803	p-Tolunitrile	1078	0.511
25	15.168	2-Methylbenzoxazole	1089	0.568
26	15.419	2,6-Dimethylphenol	1097	0.820
27	15.601	Phenylacetone	1103	0.116
28	15.698	1-Isopropyl-1-cyclohexene	1106	0.226
29	16.045	4-Ethylphenol	1117	0.681
30	16.490	Decamethylcyclopentasiloxane	1131	0.372
31	16.721	Endo-borneol	1139	2.029
32	16.918	2-Acetyltoluene	1145	0.314
33	17.065	(-)-Terpinen-4-ol	1150	2.220
34	17.262	1-(3-Methylphenyl)ethanone	1156	0.818
35	17.460	(−)-Alpha-terpineol	1163	1.438
36	17.581	(+)-Dihydrocarvone	1167	0.338
37	17.960	(+/−)-cis-piperitol	1179	0.350
38	18.027	Verbenone	1181	0.190
39	18.279	(−)-cis-carveol	1189	0.357
40	18.610	2,4-Dimethylanisole	1201	0.248
41	19.293	Piperitone	1223	0.187
42	20.994	1-Methylindan-2-one	1282	0.261
43	21.356	Dodecamethylcyclohexasiloxane	1294	0.193
44	22.485	3,3-Dimethyl-1-indanone	1336	0.233
45	23.314	Methyl eugenol	1366	0.229
46	23.669	2,3-Dimethylnaphthalene	1379	0.146
47	26.001	2,4-Di-tert-butylphenol	1468	0.644

**Table 6:** Toxic compounds in moxa smoke.

No.	Compound	CAS RN	Chemical ID	PubChem CID
1	Toluene	108-88-3	D014050	1140
2	Pyrimidine	289-95-2	C030986	9260
3	1-Methylpyrrole	96-54-8	C096654	7304
4	Pyridine	110-86-1	C023666	1049
5	Cyclopentanone	120-92-3	C007201	8452
6	1-Octene	111-66-0	C037690	8125
7	Tetrachloroethylene	127-18-4	D013750	31373
8	Octane	111-65-9	C026728	356
9	4-Aminopyridine	504-24-5	D015761	1727
10	Ethylbenzene	100-41-4	C004912	7500
11	Styrene	100-42-5	D020058	7501
12	*p*-Xylene	106-42-3	C031286	7809
13	Furfural	98-01-1	D005662	7362
14	2,5-Dimethylpyrrole	625-84-3	C067286	12265
15	2-Furanmethanol	98-00-0	C012986	7361
16	2-Acetylfuran	1192-62-7	C039669	14505
17	3-Methylpyridine	108-99-6	C053603	7970
18	2,6-Dimethylpyridine	108-48-5	C013093	7937
19	o-Xylene	95-47-6	C026114	7237
20	Butyrolactone	96-48-0	D015107	7302
21	Cyclohexanone	108-94-1	C036468	7967
22	Phenyl ethyne	536-74-3	C044736	10821
23	m-Xylene	108-38-3	C031285	7929
24	Propyl benzene	103-65-1	C024268	7668
25	Nonane	111-84-2	C017573	8141
26	2-Ethylpyridine	100-71-0	C051672	7523
27	Benzaldehyde	100-52-7	C032175	240
28	Cumene	98-82-8	C015763	7406
29	2,4-Dimethylpyridine	108-47-4	C078448	7936
30	Benzofuran	271-89-6	C105430	9223
31	5-Methylfurfural	620-02-0	C048065	12097
32	Phenol	108-95-2	D019800	996
33	Limonene	138-86-3	D000077222	22311
34	3-Ethyltoluene	620-14-4	C029719	12100
35	1,2,3-Trimethylbenzene	526-73-8	C010179	10686
36	Mesitylene	108-67-8	C010219	7947
37	Phenetole	103-73-1	C079413	7674
38	Eucalyptol	470-82-6	D000077591	2758
39	Benzene	71-43-2	D001554	241
40	Aniline	62-53-3	C023650	6115
41	Alpha-methyl styrene	98-83-9	C017915	7407
42	Butyl butyrate	109-21-7	C022793	7983
43	Decane	124-18-5	C012867	15600
44	Gamma-terpinene	99-85-4	C018669	7461
45	Alpha-phellandrene	99-83-2	C005403	7460
46	5-Ethyl-2-methylpyridine	104-90-5	C019196	7728
47	o-Cresol	95-48-7	C034047	335
48	Acrylamide	79-06-1	D020106	6579
49	2-Acetyl-5-methylfuran	1193-79-9	C057528	14514
50	*p*-Cresol	106-44-5	C032538	2879
51	1,2,4-trimethylbenzene	95-63-6	C010313	7247
52	o-Cymene	527-84-4	C046257	10703
53	*p*-Cymene	99-87-6	C007210	7463
54	Guaiacol	90-05-1	D006139	460
55	m-Cresol	108-39-4	C042041	342
56	Allylbenzene	300-57-2	C102347	9309
57	Indene	95-13-6	C093581	7219
58	Acetophenone	98-86-2	C038699	7410
59	Methyl benzoate	93-58-3	C044605	7150
60	2,6-Dimethylphenol	576-26-1	C036531	11335
61	Undecane	1120-21-4	C022884	14257
62	Phenylacetone	103-79-7	C008863	7678
63	4-Pyridinol	626-64-2	C534143	12290
64	Naphthalene	91-20-3	C031721	931
65	Azulene	275-51-4	C005525	9231
66	4-Ethylphenol	123-07-9	C042291	31242
67	4-Allyltoluene	3333-13-9	C092903	76851
68	Indolizine	274-40-8	C035094	9230
69	Phenyl acetonitrile	140-29-4	C006725	8794
70	2,3-Dimethylphenol	526-75-0	C054067	10687
71	1,2,3,4-Tetramethylbenzene	488-23-3	C021246	10263
72	(−)-Alpha-terpineol	10482-56-1	C016775	443162
73	3,5-Dimethylphenol	108-68-9	C016834	7948
74	Terpinen-4-ol	562-74-3	C034019	11230
75	Dodecane	112-40-3	C007548	8182
76	Catechol	120-80-9	C034221	289
77	5,6-Dimethylbenzimidazole	582-60-5	C015158	675
78	Tridecane	629-50-5	C094074	12388
79	(E)-Cinnamaldehyde	104-55-2	C012843	637511
80	Indole	120-72-9	C030374	798
81	Isoquinoline	119-65-3	C039109	8405
82	Citral	5392-40-5	C007076	638011
83	Hydroquinone	123-31-9	C031927	785
84	Biphenyl	92-52-4	C010574	7095
85	1-Tridecene	2437-56-1	C028691	17095
86	Tetradecane	629-59-4	C024713	12389
87	2-Methylnaphthalene	91-57-6	C027384	7055
88	1-Methylnaphthalene	90-12-0	C025968	7002
89	2-Methoxy-4-vinylphenol	7786-61-0	C014245	332
90	2,6-Dimethylnaphthalene	581-42-0	C028519	11387
91	2-Methylhydroquinone	95-71-6	C062397	7253
92	Methyl eugenol	93-15-2	C005223	7127
93	5-Methylindole	614-96-0	C093726	11978
94	2,3-Dimethylnaphthalene	581-40-8	C091753	11386
95	3-Methylindole	83-34-1	D012862	6736
96	1,4-Dimethylnaphthalene	571-58-4	C031969	11304
97	2,4-Di-tert-butylphenol	96-76-4	C056559	7311
98	Dibenzofuran	132-64-9	C023614	568
99	Phenylephrine	59-42-7	D010656	6041
100	Heptadecane	629-78-7	C016486	12398
101	Spathulenol	6750-60-3	C013258	92231
102	1-Octadecene	112-88-9	C109760	8217
103	Chamazulene	529-05-5	C013872	10719
104	Phenanthrene	85-01-8	C031181	995
105	Octadecane	593-45-3	C022883	11635
106	Pinane	473-55-2	C030216	10129
107	Nonadecane	629-92-5	C061580	12401
108	Hentriacontane	630-04-6	C049203	12410
109	Methyl palmitate	112-39-0	C019012	8181
110	Ambrettolide	123-69-3	C008563	5365703
111	Dibutyl phthalate	84-74-2	D003993	3026
112	Icosane	112-95-8	C050821	8222

## Data Availability

The data used to support the findings of this study are included within the article and in the supplementary figure. The prior studies (and datasets) are cited at relevant places within the text as references [21, 43].

## Supplementary Materials

Supplementary Materials Supplementary Figure S1: microscopic observations of heart, liver, and kidney pathology.
