# Supplementary material for: Analysis of the Components in Moxa Smoke by GC-MS and Preliminary Discussion on Its Toxicity and Side Effects
Source: Evid Based Complement Alternat Med. 2020 Oct 31;2020:2648759. doi: 10.1155/2020/2648759 (PMC7648687; doi:10.1155/2020/2648759)
Supplement: Supplementary Materials — Supplementary Figure S1: microscopic observations of heart, liver, and kidney pathology. [file 2648759.f1.pdf]

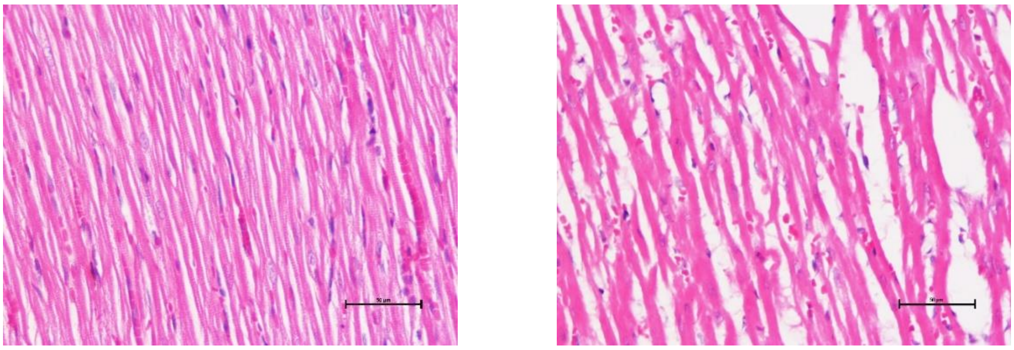

Control group Moxa smoke group

(a) Microscopic observation of heart pathology (HE stain, ×40).

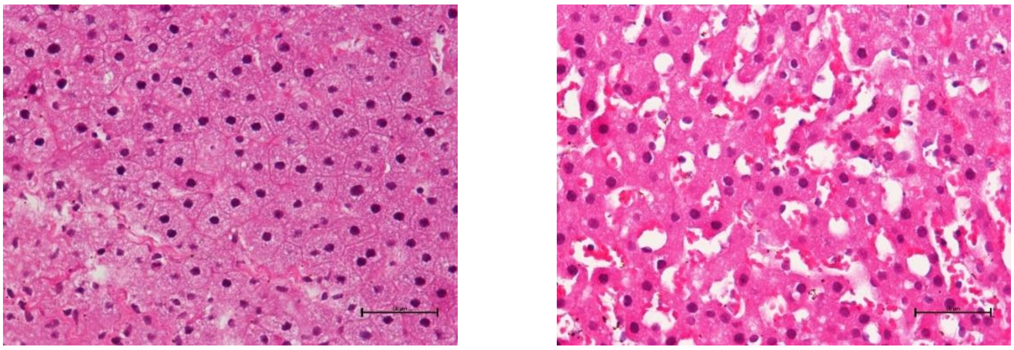

Control group Moxa smoke group

(b) Microscopic observation of liver pathology (HE stain, ×40).

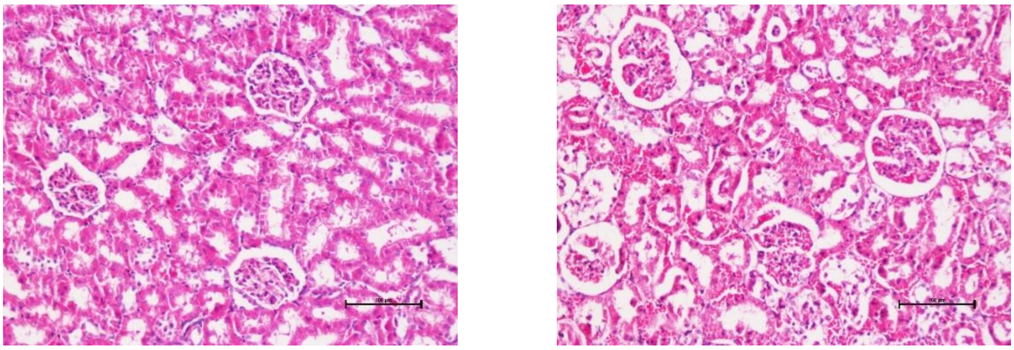

Control group Moxa smoke group

(c) Microscopic observation of kidney pathology (HE stain, ×20).

Supplementary Figure S1. Microscopic observations of heart, liver, and kidney pathology.
